# Performance of a Distributed Simultaneous Strain and Temperature Sensor Based on a Fabry-Perot Laser Diode and a Dual-Stage FBG Optical Demultiplexer

**DOI:** 10.3390/s131115452

**Published:** 2013-11-12

**Authors:** Suhwan Kim, Hyungwoo Kwon, Injae Yang, Seungho Lee, Jeehyun Kim, Shinwon Kang

**Affiliations:** 1 School of Electronics Engineering, College of IT Engineering, Kyungpook National University, 1370 Sankyuk-dong, Buk-gu, Daegu 702-701, Korea; E-Mail: shkim@ee.knu.ac.kr; 2 School of Electrical Engineering and Computer Science, Kyungpook National University, 1370 Sankyuk-dong, Buk-gu, Daegu 702-701, Korea; E-Mail: geetoo@naver.com; 3 Mine Reclamation Corporation, Chungjindonggil 30, Jongro-gu, Seoul 110-727, Korea; E-Mails: ygloria@mireco.or.kr (I.Y.); cortoimasa@mireco.or.kr (S.L.)

**Keywords:** Fabry-Perot laser diode, OTDR, BOTDR, distributed optical sensors, simultaneous temperature and strain measurement

## Abstract

A simultaneous strain and temperature measurement method using a Fabry-Perot laser diode (FP-LD) and a dual-stage fiber Bragg grating (FBG) optical demultiplexer was applied to a distributed sensor system based on Brillouin optical time domain reflectometry (BOTDR). By using a Kalman filter, we improved the performance of the FP-LD based OTDR, and decreased the noise using the dual-stage FBG optical demultiplexer. Applying the two developed components to the BOTDR system and using a temperature compensating algorithm, we successfully demonstrated the simultaneous measurement of strain and temperature distributions under various experimental conditions. The observed errors in the temperature and strain measured using the developed sensing system were 0.6 °C and 50 με, and the spatial resolution was 1 m, respectively.

## 1. Introduction

Because of their merits such as wide bandwidth and stability to electromagnetic induction, optical fibers can overcome the limitations of traditional coaxial cables and therefore, optical fibers have been widely used for super-high-speed communication in the past few decades. Recently, there have been many studies on transmission media for communication as well as on sensor systems that remotely measure strain or temperature by analyzing reflected or backscattered light intensity [1–3]. Optical fiber is a passive sensing material that does not consume electrical power. Its durability enables a long time measurement of the target object, and its stability to electromagnetic interference guarantees high reliability.

Compared with optical fiber sensor systems using Raman backscattering, distributed systems using Brillouin scattering have low threshold power as well as the ability to simultaneously measure temperature and strain. Thus, many studies of various distributed measurements using spontaneous and stimulated Brillouin scattering have been undertaken. The Brillouin Optical Time Domain Reflectometer (BOTDR) is a system that uses spontaneous scattering to measure the return time of optical pulses entering into a fiber under test (FUT) and the intensity of scattered light at each sampling point. By analyzing the measured time and the intensity, the BOTDR calculates distributed physical quantities such as temperature and strain [4–9]. Because this method, which uses only one side of the FUT, is single-ended, it is suitable for long-range remote measurements as well as the simultaneous measurement of temperature and strain. However, this system detects signals below 40–50 dB, which are weak compared to the entering optical pulse, therefore, post processing such as averaging and digital filtering is required. This necessitates a longer measurement time, but the measurement time was dramatically shortened recently because various solutions for high-speed sampling were commercialized.

In general, there are two methods to compensate for the extrinsic loss of the BOTDR system for the simultaneous measurement of temperature and strain. In the first method, the absolute temperature of the FUT is measured in a constant temperature environment and is used for the normalization of the reference signal. The second method consists of measuring both the Brillouin OTDR and a Rayleigh OTDR from the FUT and normalizing the BOTDR using the Rayleigh OTDR measurement. The first method gives a smoother shape than the second method because it measures the same FUT twice. However, it is difficult to use this method in long-range measurement, e.g., over a few kilometers or several connected optical fibers, because Brillouin OTDR takes more time than Rayleigh OTDR.

In this paper, we propose and demonstrate a BOTDR system employing a low-cost FP-LD, which effectively compensates for extrinsic loss, and a dual-stage FBG optical demultiplexer for noise reduction. Instead of an erbium-doped Q-switch laser or a superluminescent light emitting diode (SLED), which are generally used for BOTDR [10,11], we applied the low-cost FP-LD to a Rayleigh OTDR and used the resulting measurement for normalizing the Brillouin OTDR. We compared the performance of the FP-LD based Rayleigh OTDR with that of one based on a SLED. After applying the FP-LD to the BOTDR system, we confirmed the system's simultaneous measurement of strain and temperature. In order to improve the noise characteristics of the Brillouin scattering signal in our BOTDR system, we developed a dual-stage FBG optical fiber demultiplexer, applied it to the BOTDR system, and verified its characteristics.

## 2. Materials and Method

### 2.1. Dual-Stage FBG Optical Demultipelxer for Reducing the Rayleigh Noise

Brillouin scattered lights are separated by as much as 11 GHz (approximately 90 pm in the wavelength domain), so it is difficult to separate them using a general optical thin film filter. In previous studies, researchers used methods that adjust the free spectral range (FSP) of a Fabry-Perot or Mach-Zehnder interferometer to be equal to the separation frequency [2,12]. The FP interferometer has the advantage of very narrow transmittance bandwidth, but its signal to noise ratio (SNR) is quite low because of high insertion loss. When using the Mach-Zehnder optical fiber interferometer, the insertion loss is low, but it is difficult to control the difference between the optical paths for configuring the FSR and securing the stability because the two optical paths are very sensitive to the temperature and polarization status.

**Figure 1.**
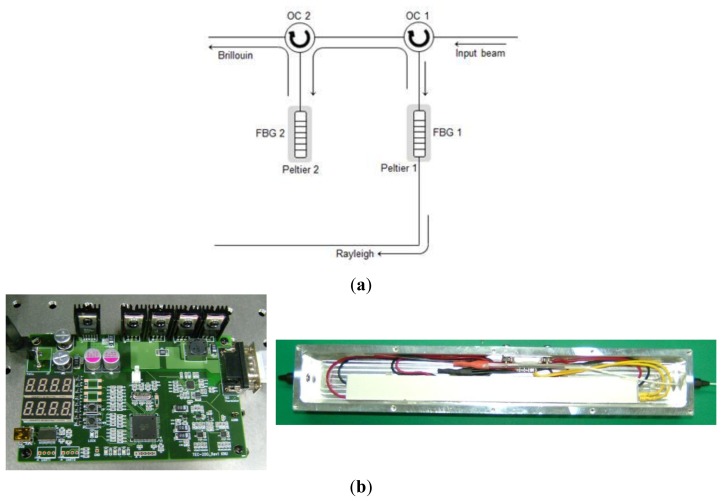
(**a**) The schematic diagram and (**b**) photograph of fabricated dual-stage FBG optical demultiplexer and temperature control device.

To address these flaws, we developed the dual-stage FBG optical demultiplxer. The schematic diagram and photograph of the fabricated dual-stage FBG optical demultiplexer and temperature control device for a Peltier device are shown in Figure 1. With the optical multiplexer using two FBG filter as a passive optical fiber device, we ensure low insertion loss and high selectivity through multi-fabrication with an optical circulator. We also applied the Peltier module for changing the temperature to control the center wavelength of the FBG.

First, the center wavelengths of the FBG 1 and 2 were fixed to the Brillouin anti-Stokes wavelength by controlling the temperature of the Peltier devices. The anti-Stokes component from the light signal enters the optical circulator (OC) 1 is reflected from the FBG 1 and enters into the OC 2, and the Rayleigh component is detected at the photodetector after transmitting through the FBG 1. The Rayleigh component of the anti-Stokes component reflected from the FBG 1 is removed again at FBG 2. Thus, the Brillouin and Rayleigh components can be propagated through independent optical paths.

### 2.2. OTDR Using the FP-LD

**Figure 2.**
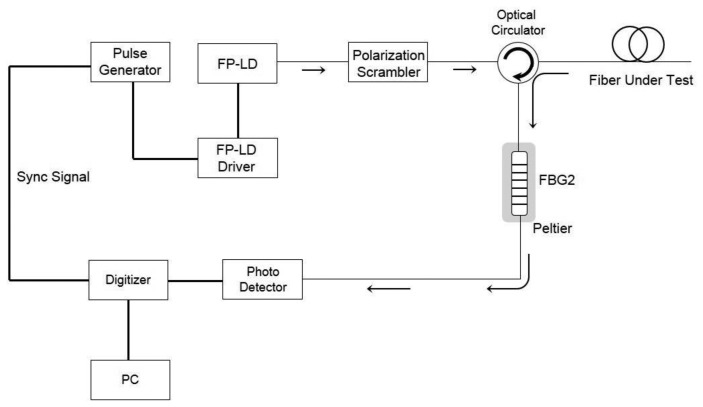
Schematic diagram of Rayleigh OTDR.

The power of Brillouin scattered light changes by 0.3% with respect to temperature change. To separate the intensity component contributing to temperature change in the direct detection system that detects the intensity of the scattered light and calculates the temperature, the power attenuation in the optical fiber due to the connection, bending, or distance must be compensated. Because the Rayleigh scattered light has little responsiveness to temperature and strain, it can generally be used as a compensating method for direct detection in the distributed optical fiber sensor system. A schematic of the developed Rayleigh OTDR is shown in Figure 2.

**Figure 3.**
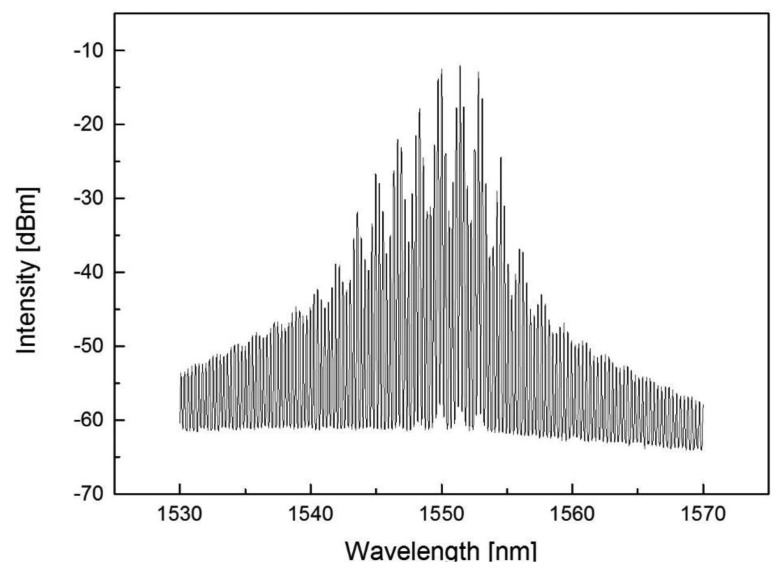
The spectrum of the FP-LD used for Rayleigh OTDR.

An FP-LD with a center wavelength of 1,550 nm (Figure 3) along with an Avalanche Photodiode (APD) as a photodetector is used. After measuring the temperature of the APD using a thermistor, we set the proper bias voltage of the APD using a Digital Analog Convertor (DAC). Subsequently, optical pulse signals are introduced into the FUT by using the pulse signal with an LD switch. After converting into current, backscattered light is sequentially punched into the APD and log convertor. The log convertor converts the input current to the log scale and output the analog results, and the microprocessor unit (MCU) performs the averaging of the DAC results.

### 2.3. Configuration of the BOTDR System

**Figure 4.**
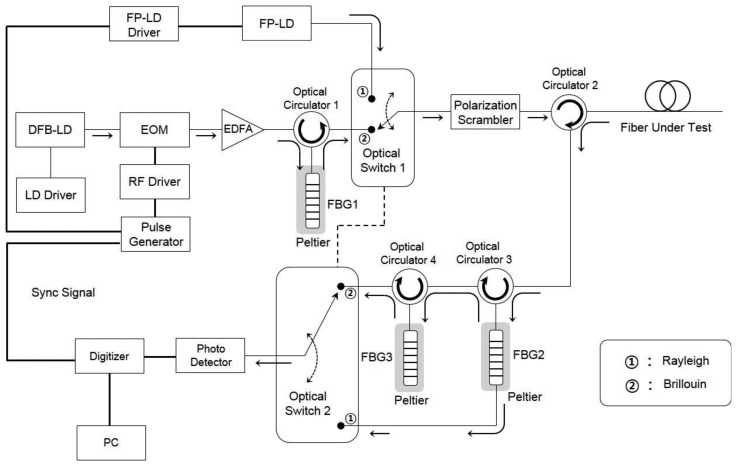
Schematic diagram of the developed OTDR and BOTDR systems.

The Brillouin OTDR is the main optics in the distributed optical fiber sensor. It collects the backscattered Brillouin signal from the optical pulse in the FUT and measures the intensity proportional to the temperature and strain change in the time domain. The developed BOTDR system shown in Figure 4 uses a distributed feedback laser diode (DFB-LD) as a light source and generates the optical pulse through the external modulator method using an electro-optic modulator (EOM). External modulation turns the DFB-LD on and off with an extinction ratio of over 40 dB by modulating the EOM using an RF driver with a TTL signal from a pulse generator. The generated optical pulse is amplified to 500 mW using the Erbium-Doped Fiber Amplifier (EDFA), and its amplified spontaneous emission (ASE) noise is removed using FBG 1. The Rayleigh scattered light components of backscattered light from the FUT are removed using the developed dual-stage FBG optical demultiplexer (FBG 2 and 3). The Rayleigh light component is acquired from the developed Rayleigh OTDR on the basis of the FP-LD. We then measure the distributed temperature from the normalized intensity, which is the Brillouin to Rayleigh power ratio.

Besides along with the direct detection method to measure the temperature, we developed the coherent detection method, which identifies the distributed temperature and strain by measuring the frequency shift of anti-Stokes light and calculating the temperature and strain proportional to the frequency shift, through the preceding study [13]. We fabricated the direct and coherent detection systems together, applied the temperature compensation algorithm to the developed system, and completed the distributed optical fiber sensor system for simultaneous strain and temperature measurements. We applied a well-known temperature compensation algorithm described by Equations (1) and (2). We used the parameters K_ν_^T^ and K_ν_^ε^ as the temperature and strain coefficients with values of 1.1 MHz·°C^−1^ and 5 MHz/100 με, respectively, and K_P_^T^ and K_P_^ε^ as the coefficients for power variations with respect to temperature (0.3%·°C^−1^) and strain (−0.09%/100 με), respectively [14]:

(1) $$\Delta T=\frac{K_{\varepsilon }^{\upsilon }\Delta P-K_{\in }^{P}\Delta \upsilon }{K_{\varepsilon }^{\upsilon }K_{T}^{P}-K_{\varepsilon }^{P}K_{T}^{\upsilon }}$$

(2) $$\Delta \varepsilon =\frac{K_{T}^{P}\Delta \upsilon -K_{T}^{\upsilon }\Delta P}{K_{\varepsilon }^{\upsilon }K_{T}^{P}-K_{\varepsilon }^{P}K_{T}^{\upsilon }}$$

## 3. Results and Discussion

### 3.1. Properties of the Fabricated Dual-Stage FBG Optical Demultiplexer

**Figure 5.**
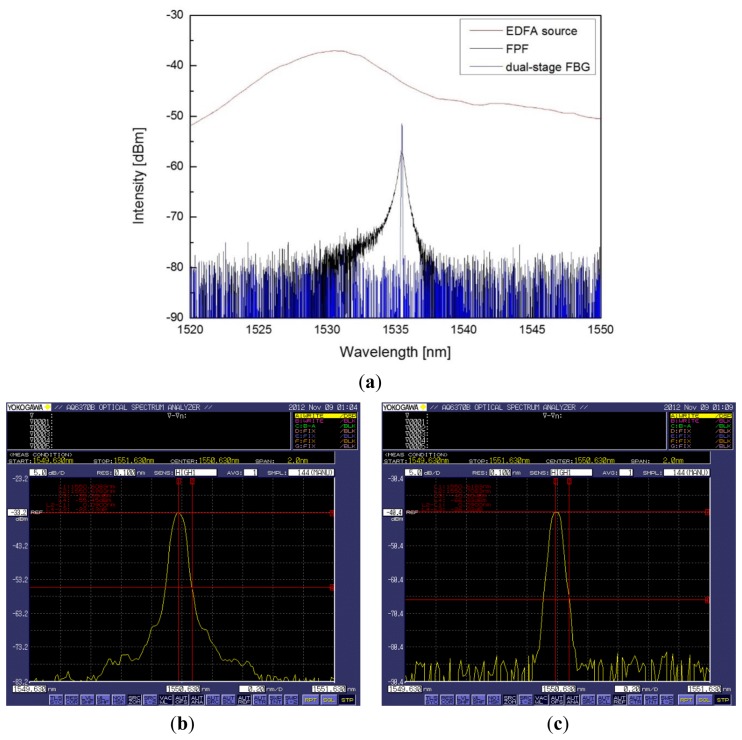
Optical spectrum of (**a**) comparison the dual-stage FBG demultiplexer with FPF (λ_0_ = 1,535.47 nm), (**b**) the single-stage FBG, and (**c**) the dual-stage FBG.

We separated the Brillouin component from the FBG reflection light by fixing the center wavelength of the FBG to the Brillouin anti-Stokes, but it is possible to fix the center wavelength of FBG to Rayleigh to separate the Brillouin component. In that case, more attention is required for the cavity interference from the etalon because of the direct connection of two FBG filters. Optical characteristics of the fabricated optical demultiplexer with the Fabry-Parot Filter (FPF) are compared and listed in Table 1 and the spectrum comparison result is shown in Figure 5. Compared with the FPF, the insertion loss was improved by as much as 6.1 dB and 0.5 dB bandwidth is improved by 10 times. The separation ratio of Rayleigh and Brillouin components was 22 dB when using the single-stage structure, but it is improved by as much as 4 dB when using the dual-stage structure.

**Figure 6.**
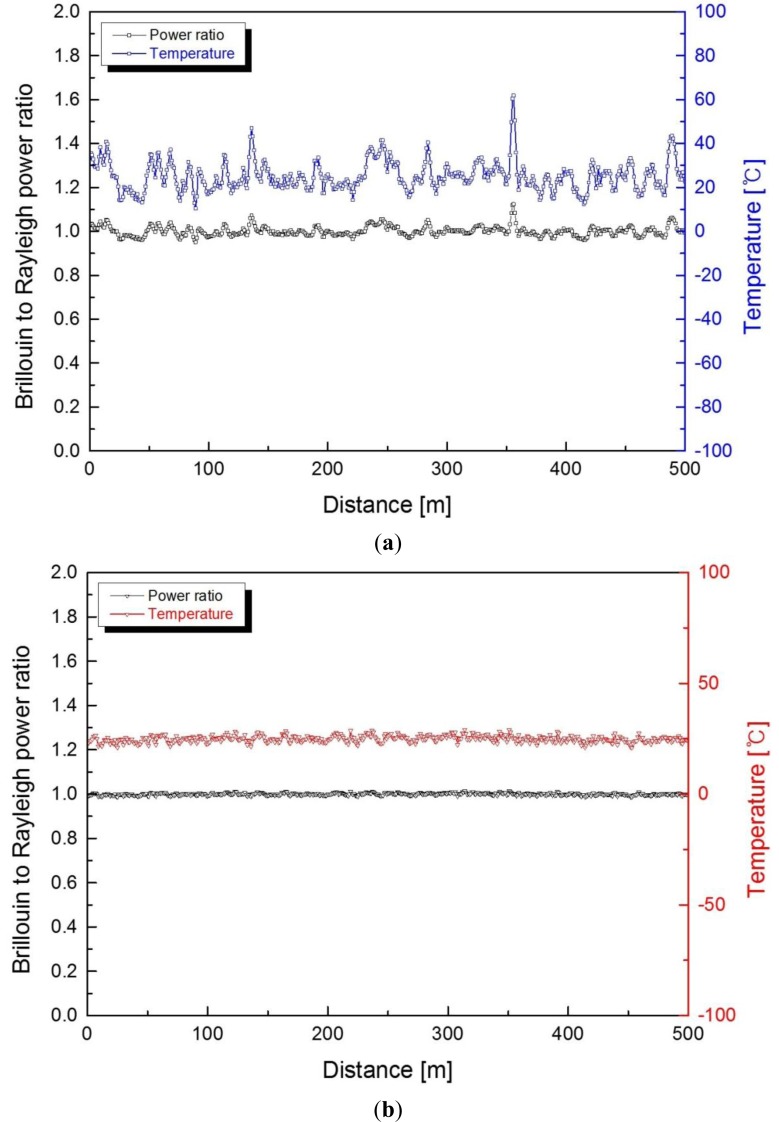
Measured Brillouin to Rayleigh power ratio and distributed temperature profiles for (**a**) the single-stage and (**b**) the dual-stage FBG demultiplexer.

Figure 6 shows the ratio of Brillouin to Rayleigh intensities and distributed temperature profiles for both types of FBG demultiplexers from the 500 m optical fiber at room temperature. On comparing the temperature results of the one-stage FBG with those of the dual-stage FBG optical demultiplexer, the former shows a higher standard deviation as much as 5.32 °C because of the correlation Rayleigh noise from the remaining Rayleigh scattered light. The temperature resolution of the system can deteriorate significantly if the Rayleigh scattered light is not removed thoroughly.

### 3.2. Characteristics of OTDR Using the FP-LD

**Figure 7.**
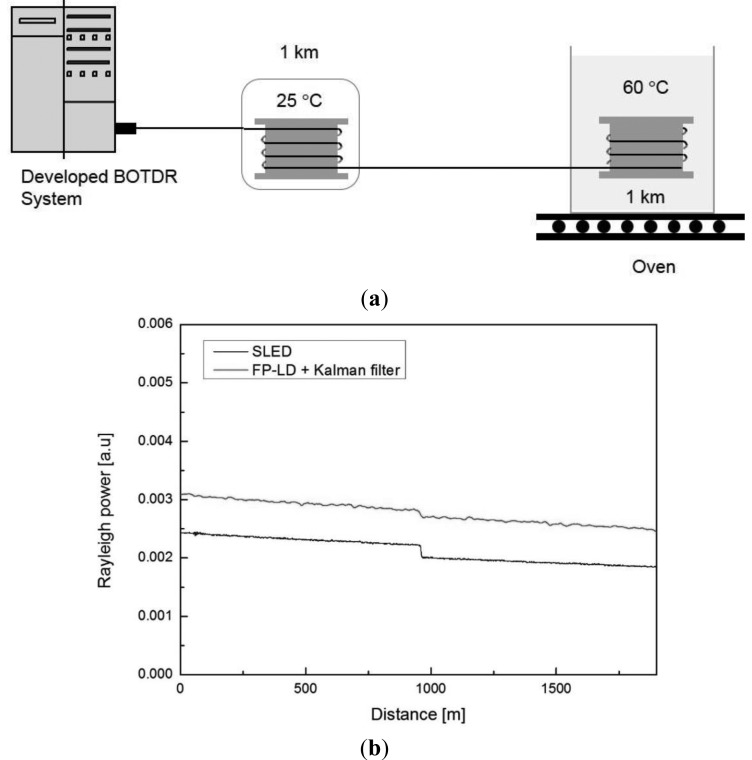
(**a**) Experimental setup and (**b**) comparison of FP-LD and SLED based OTDR trace for evaluating the performance.

First, we confirmed the applicability of Rayleigh OTDR using the FP-LD as the reference waveform of the distributed optical fiber sensor system. The Rayleigh OTDR trace was measured using a 10 ns laser pulse from the FUT of two fusion spliced 1-km-long optical fibers (Figure 7a). The averaging number was 256,000 and the sampling rate was 200 MHz. A Kalman filter was applied to the measurement result to improve the characteristics. The results were then compared with the case of using the SLED as a light source. As shown in Figure 7b, the system could detect the power loss due to the fusion splice at the same point. To analyze the noise characteristics of the two OTDR systems, the RMSE values were compared. We observed a maximum RMSE value of 0.000242 and 0.000249 for the SLED and Kalman filter applied FP-LD based OTDR trace, respectively. And a maximum RMSE value of 0.0002 and an averaging value of 0.000011 when we compared with two OTDR traces. On the basis of these results, we confirm that the Rayleigh OTDR based on the FP-LD and Kalman filter can be effectively used for compensating extrinsic loss of the developed BOTDR system.

### 3.3. Performance Evaluation of the Developed BOTDR System

**Figure 8.**
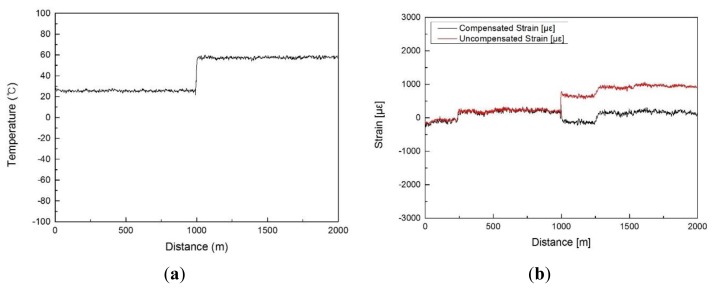
Distributed (**a**) temperature and (**b**) strain profiles measured from the developed BOTDR system.

We evaluated the performance of the developed BOTDR system by employing an FP-LD based OTDR, a dual-stage FBG optical demultiplexer, and a coherent detection method. First, the Rayleigh and Brillouin OTDRs were measured and the Brillouin OTDR result was normalized to Rayleigh OTDR as a reference wave. Then, the coherent detection results were acquired according to the frequency change. As in the preceding experiment for testing the Rayleigh OTDR, two 1-km-long optical fibers were used. In this experiment, however, the second optical fiber spool was placed in a constant temperature oven at 60 °C before we simultaneously measured the temperature and strain. In the case of the distributed temperature measurement, we confirmed the controlled temperature at the optical fiber as shown in the Figure 8a. From the FP-LD based Rayleigh OTDR and Brillouin power measurements, a temperature resolution of approximately 3 °C was secured. In the distributed strain measurement shown in Figure 8b, the natural strain from bending the bobbin was precisely measured using the temperature compensation algorithm. We calculated the compensated strain by applying the Rayleigh OTDR and Brillouin power to the uncompensated strain (frequency shift) measured by the coherent detection method. In the temperature experiment, the maximum temperature measurement uncertainty of the 2 km fiber increased from −1.59 °C to 1.69 °C and the average error value was approximately 0.6 °C. In the strain experiment, the maximum strain uncertainty increased from −278 to 325 με and the average error value was approximately 50 με. The data was taken with 10 ns launched pulses, which corresponds to 1 m spatial resolution and the measurement time was as low as 1 min 30 s.

**Figure 9.**
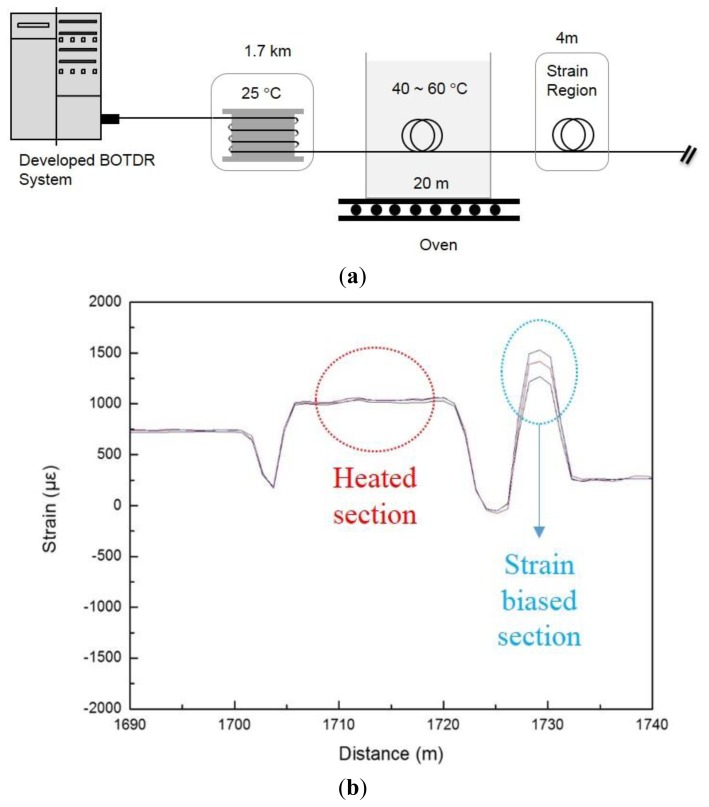
(**a**) Experimental setup for evaluating the strain measurement performance of the developed Brillouin OTDR system and (**b**) the result of the strain change profiles measurement.

For more detailed testing of the performance of simultaneous measurement, we conducted additional experiments. First, a 1.7-km-long optical fiber was kept at room temperature; subsequently, a 20-m region was placed in the temperature oven at a temperature of 40–60 °C with free strain. The 4-m regions at both ends of the fiber were fixed to the micro-stage, and we applied the strain change in that region as shown in Figure 9a. To increase the strain, the micro-stage was moved by 0.127 mm. so that the center part of the 4-m region was tensed gradually. As a result, there was no strain response with respect to the temperature change in the strain-free heated section, and 120 με of the increased strain was completely measured according to the tension, as shown in Figure 9b.

## 4. Conclusions

A BOTDR sensor system for the simultaneous measurement of temperature and strain, based on an FP-LD applied OTDR and dual-stage FBG optical demultiplexer, has been proposed and experimentally demonstrated. We compared the performance of Rayleigh OTDR based on the FP-LD and an SLED light source, secured an RMSE value of 0.2%, and proved its applicability to a distributed optical fiber sensor system as a reference wave. To overcome the demerits of the traditional Fabry-Perot and Mach-Zehnder interferometers, we fabricated a dual-stage FBG optical demultiplexer for improving the noise characteristics of the developed system. By fabricating the dual-stage optical demultiplexer with 0.06 nm passband and controlling the temperature using the Feltier module, we improved the separation ratio of Rayleigh and Brillouin by as much as 4 dB. After applying these results to the BOTDR system with a temperature compensation algorithm and a coherent detection method, we evaluated the performance of the developed BOTDR. The simultaneous measurement of temperature and strain was successfully performed within maximum measurement error of 1.6 °C for temperature, 325 με for strain and 1-m spatial resolution was secured from the 10 nm pulse signal. These results show a considerable improvement over previously published work on the use of BOTDR for simultaneous measurement of temperature and strain. Furthermore, replacing the expensive SLED with the FP-LD in the BOTDR system results in a substantial reduction in cost.

## Figures and Tables

**Table 1.** Optical properties comparison of the fabricated FBG optical demultiplexer with FPF.

**Property**	**Developed Demultiplexer**	**FPF**
Center wavelength	1,535.47 nm	1,535.47 nm
0.5 dB bandwidth	0.1 nm	1.1 nm
Insertion loss	2.1 dB	8.2 dB
